# Electromagnetic interactions in regulations of cell behaviors and morphogenesis

**DOI:** 10.3389/fcell.2022.1014030

**Published:** 2022-10-19

**Authors:** Guogui Sun, Jiong Li, Wei Zhou, Rosalie G. Hoyle, Yue Zhao

**Affiliations:** ^1^ School of Public Health, North China University of Science and Technology Affiliated People’s Hospital, North China University of Science and Technology, Tangshan, China; ^2^ Department of Medicinal Chemistry, Virginia Commonwealth University, Richmond, VA, United States; ^3^ Massey Cancer Center, Philips Institute for Oral Health Research, Virginia Commonwealth University, Richmond, VA, United States; ^4^ Cancer Hospital of University of Chinese Academy of Sciences (Zhejiang Cancer Hospital), Institute of Basic Medicine and Cancer of Chinese Academy of Sciences, Hangzhou, China

**Keywords:** centrosome, cellular electric field, nano-electronic generator, electric oscillator, microtubules, transmembrane potential

## Abstract

Emerging evidence indicates that the cellular electromagnetic field regulates the fundamental physics of cell biology. The electromagnetic oscillations and synchronization of biomolecules triggered by the internal and external pulses serve as the physical basis of the cellular electromagnetic field. Recent studies have indicated that centrosomes, a small organelle in eukaryotic cells that organize spindle microtubules during mitosis, also function as a nano-electronic generator in cells. Additionally, cellular electromagnetic fields are defined by cell types and correlated to the epigenetic status of the cell. These interactions between tissue-specific electromagnetic fields and chromatin fibers of progenitor cells regulate cell differentiation and organ sizes. The same mechanism is implicated in the regulation of tissue homeostasis and morphological adaptation in evolution. Intercellular electromagnetic interactions also regulate the migratory behaviors of cells and the morphogenesis programs of neural circuits. The process is closely linked with centrosome function and intercellular communication of the electromagnetic fields of microtubule filaments. Clearly, more and more evidence has shown the importance of cellular electromagnetic fields in regulatory processes. Furthermore, a detailed understanding of the physical nature of the inter- and intracellular electromagnetic interactions will better our understanding of fundamental biological questions and a wide range of biological processes.

## Introduction

For decades, biologists have been trying to figure out the underlying physical mechanisms for the self-organization of super macromolecules and organelles within a cell, also known as “order from order” (Mentre, 2012). Despite the tremendous progress the field has made in understanding the molecular basis of cellular events in the past decades, a single cell’s omnipotent ability for self-organization, adaptation, and evolution is still a mystery. Cellular consciousness models have emerged to provide wholistic views of cellular electromagnetic interactions within and between the cellular protein complexes, nucleic acids, and transmembrane electric currents (De Loof, 2016; Baluska et al., 2021; Timsit and Gregoire, 2021). Like a nano-brain for the cell, the immaterial and protean nature of such interactions are capable of processing and integrating the vast amounts of environmental cues at nanoscopic scales and eventually orchestrated in the kaleidoscopic programs of transformations in morphogenesis and evolution.

Bioelectricity regulated by ion pumps and ion channels, which maintains the membrane potential of cells, also plays important roles in stem cell differentiation and embryo development (Levin, 2021). The crosstalk between transmembrane potentials and intracellular electromagnetic interactions may represent interesting areas of research to unveil this mystery of cells. From the quantum biology point of view, electromagnetic interactions and photonic communications at intracellular and intercellular levels are indispensable for the emerging evolutions of eukaryotic cells and metazoan species (Albrecht-Buehler, 2005; Cantero et al., 2018). Microtubules and chromatin fibers are well-known electromagnetic oscillators in eukaryotic cells (Zhao and Zhan, 2012a; Zhao and Zhan, 2012b; Polesskaya et al., 2018). The centrosome and cilium in eukaryotic cells function as a nano-sized molecular electronic generator that continuously fuels the microtubule network with electric currents, generating the electromagnetic field that facilitates mitosis (Nygren et al., 2020). Chromatin electromagnetic oscillations are triggered by the energy-consuming movements of DNA/RNA polymerases and cytoskeleton electronic pulses transmitted to chromatin fibers through microtubules, which are generated by centrosome and the cytoskeleton bond ATPases (Zhao and Zhan, 2012a; Pliss et al., 2013; Niekamp et al., 2019). These electromagnetic interactions govern a plethora of cellular functionalities from gene transcriptional regulation to tissue morphogenesis.

## The electronic generator function of a centrosome

A centrosome is a small membrane-less organelle of eukaryotic cells. It is essential for mitosis and other fundamental cellular functionalities (Bettencourt-Dias and Glover, 2007). The centrosome was first discovered in the late 19th century (Nygren et al., 2020), but the structural features of the centrosome remained obscure for years due to its small size, which is usually only a few hundred nanometers in diameter (Conduit et al., 2015). However, the new generation of super-resolution microscopes has captured the molecular details of centrosomes (Chong et al., 2020). A centrosome is composed of two centrioles and pericentriolar amorphous structures, namely, pericentriolar material (PCM). In most cases, each centriole is composed of nine microtubule triplets arranged in a barrel structure. Interestingly, the two centrioles are arranged in an orthogonal configuration. Despite advancements in the structural features of centrosomes, few mechanistic insights have been concluded due to the unique super macro molecular complex in eukaryotic cells until recently.

**FIGURE 1:**
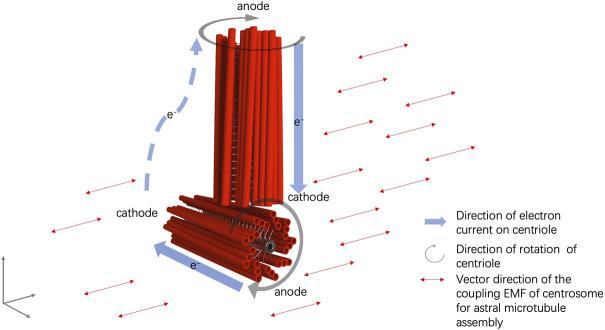
Illustration of the electromagnetic field and electric currents within and around the centrosome.

Based upon the structural details of centrosomes in combination with the latest advancements in quantum biology, it is reasonable to speculate that centrosomes function as a nano-sized electric generator in live cells (Nygren et al., 2020). Mechanistically, the dipolar structure of α and β tubulins of microtubules allows the generation of dipolar oscillations under the intracellular pulses (Zhao and Zhan, 2012a). The motor proteins within the centriole, lead to the synchronized oscillation of microtubules in the centrosome and electric excitation of the centrioles (Nygren et al., 2020). The electric excitation of centrioles generates a dynamic electromagnetic field around the microtubule triplets (Figure 1).

**FIGURE 2:**
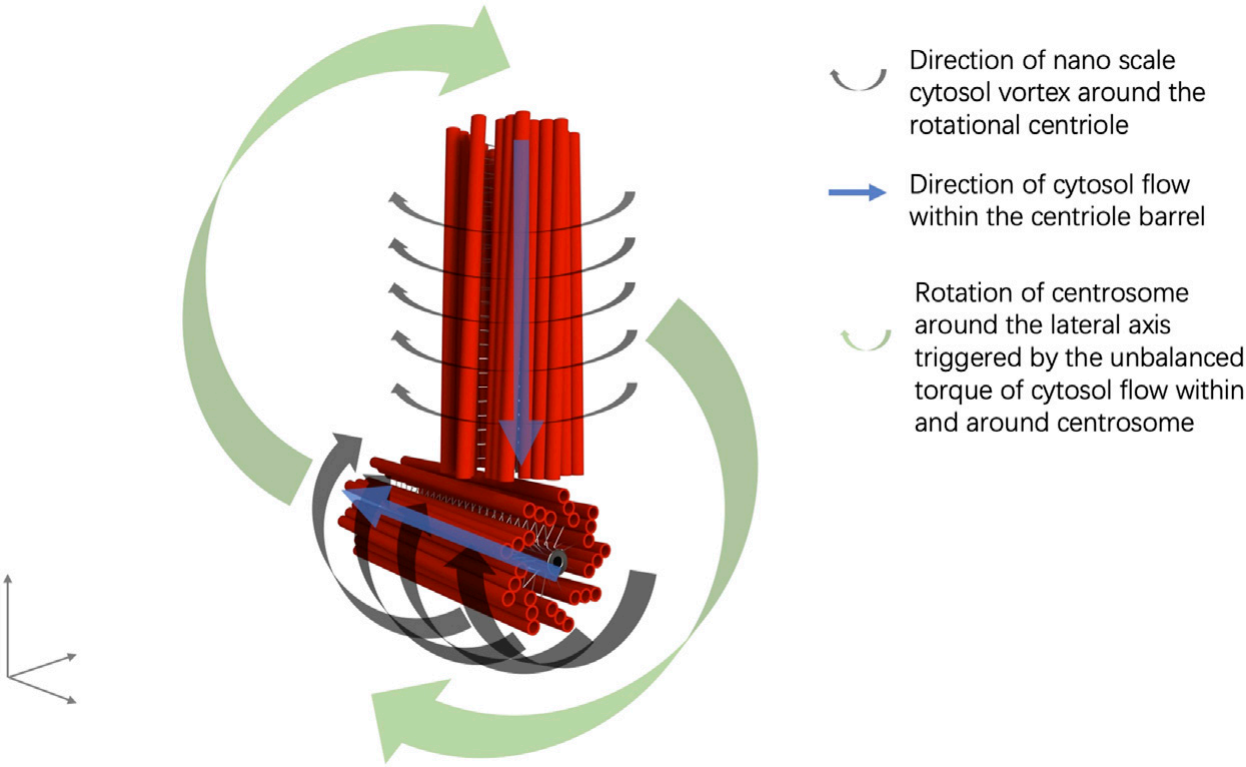
Illustration of centrosome rotation in correlation with cytosol flow.

Furthermore, the perpendicular configuration of two centrioles in a centrosome can be explained by the interaction of the EMFs of the mother and daughter centrioles, in which the rotational momentum of a centriole generates an electrical current alongside the microtubule triplets and a coupling EMF, aligned with the EMF of the other rotating centriole (Nygren et al., 2020). Thus, an electric circuit is formed from one end to the other end of the centriole, in which an anode is formed at one end of the centriole and a cathode is formed at the other end of the centriole. The cathode end of the mother centriole moves to the proximity of the anode end of the daughter centriole in the orthogonal configuration by EMF interaction (Figure 1). The anodal end of the mother centriole and the cathodal end of the daughter centriole is mutually attracted to each other. An electric circuit is also formed in the cytosol of the pericentriolar material connecting the two far ends of the mother and daughter centrioles (Figure 1). Additionally, the spinal rotation of the mother and daughter centrioles accompanied by the nano-scale cytosol flow triggered by the rotations of centrioles generates an imbalanced torque around the centrosome core structures and results in the rotation of the centrosome complex in the cytosol (Nygren et al., 2020) (Figure 2).

**FIGURE 3:**
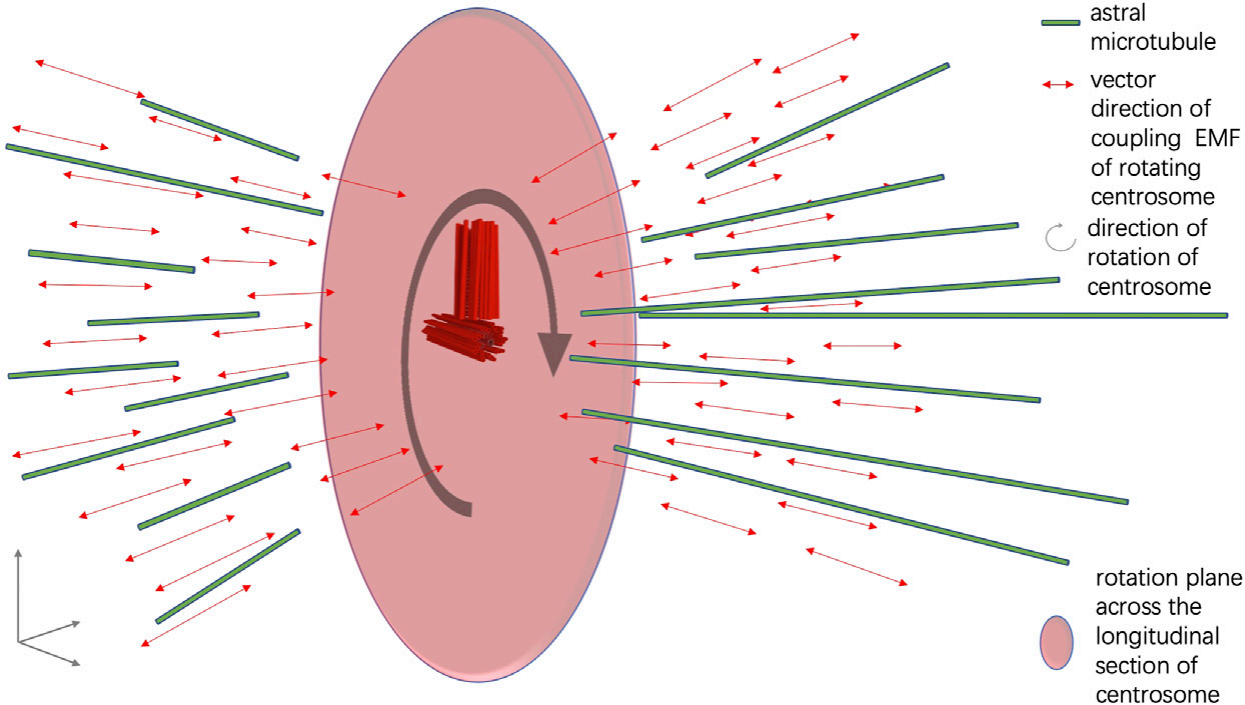
Illustration of the assembly of spindle microtubules orchestrated by EMF of the centrosome.

Nano-sized electric pumping is known to facilitate the oligomerization of α and β tubulins and promote the assembly of microtubule fibers (Sahu et al., 2014). From the quantum biology point of view, the electric current and EMF formed around the centrosome complex are important for the assembly of spindle microtubules in mitosis. When the two distal ends of the mother and daughter centriole rotate around the two proximal ends of the mother and daughter centriole, a coupling EMF is formed in the vector that is perpendicular to the longitudinal section of the mother and daughter centrioles. It is likely that the coupling EMF perpendicular to the longitudinal section of the centrosome promotes the assembly of astral microtubules in mitosis (Figure 3). The directional preferences of the spindle body microtubules are also achieved through EMF interaction between the centrosome and the intracellular EMF landscape, which is shaped by the pre-existing cytoskeleton network and chromosomes in the nucleus of a dividing cell (Zhao and Zhan, 2012a; Zhao and Zhan, 2012b).

## Cell type specificity of cellular electromagnetic fields

In metazoans, cells need to differentiate into specific lineages to form different tissues and organs to perform complicated functions and morphogenic programs. Cells of the same lineage inherit the same transcriptional programs and epigenetic transcriptional state (Hemberger et al., 2009). Theoretically, cells of a specific lineage possess specific fingerprints of dielectric frequencies (Zhao and Zhan, 2012b; Polesskaya et al., 2018). Additionally, cell type-specific dielectric fingerprints have been observed in different types of cancer cells (Barbault et al., 2009; Jafari and Hasanzadeh, 2020). During embryogenesis, the spatial and temporal proliferation and differentiation of cells result in programmed changes of cellular electromagnetic fields in different parts of the embryo to form organs, in terms of dielectric oscillatory frequencies and bioelectricity of transmembrane potentials (Levin, 2021). The progressed changes of cellular electromagnetic fields of different types of cells within a developing embryo concur with spatial and temporal changes of the subnuclear organizations of chromatin fibers, which further orchestrate the morphogenetic programs of the embryo to regulate the sizes and shapes of organs.

Liquid−liquid phase separation (LLPS) is the physical phenomenon that has been recently acknowledged as an important regulatory mechanism of gene transcription, which dynamically regulates the subnuclear compartmentations of transcription factors and chromatin regions (Hyman et al., 2014; Boija et al., 2018). Different transcriptional programs are composed of different chromosome clustering and epigenetic states of chromatin fibers in a particular liquid phase. The process is accompanied by changes in subnuclear chromatin organizations (Yuan et al., 2020). These changes further result in alterations of frequencies of the electric oscillatory chromatin subunits. Certain transcriptional factors, such as YAP and TAZ, have proven to be able to initiate specific transcriptional programs and the process of LLPS of chromatin fibers in the nucleus (Cai et al., 2019; Lu et al., 2020). Recent discoveries indicated that LLPS is ubiquitous in regulating cellular events from signal transductions to gene transcriptional regulations (Mehta and Zhang, 2022). Thus, cells with a particular epigenetic status share identical cellular electromagnetic frequencies derived from nuclear chromatin fibers, RNA molecules, and proteins.

## Synchronization of cellular electromagnetic fields

The synchronized dipolar movements of α and β tubulins in the microtubules and histones in the chromosomes are recognized as the molecular basis of the electromagnetic oscillations of microtubules and chromatin fibers (Zhao and Zhan, 2012a; Polesskaya et al., 2018). DNA/RNA molecules are also electromagnetic oscillators based upon the synchronized longitudinal oscillations of electrons in the hydrogen bonds within the DNA/RNA sequences induced by the pulses within live cells (Savelev and Myakishev-Rempel, 2020). From the physical point of view, any polymeric biomolecules, including most proteins and RNAs and given the complexity and flexibility of various types of chemical and hydrogen bonds existing within these molecules, can be viewed as electromagnetic oscillators in the live cell (Sponer et al., 2001; Zhang et al., 2021). During transcription, the electromagnetic oscillations of the transcribed chromatin fibers are transmitted to the RNA molecules transcribed. The electromagnetic oscillations of mRNAs are further passed down to tRNAs and proteins through the process of translation. Thus, the electromagnetic oscillation pattern of a particular chromatin state in the nucleus propagates in the cell through the central dogma. In such a paradigm, the cell generates a cellular electromagnetic field with patterns of electromagnetic frequencies that echo electromagnetic oscillations of the chromatin fibers in the nucleus of the cell.

## Cellular electromagnetic field in regulating organ morphogenesis

The cellular electromagnetic field plays a key role in the spatial and temporal regulations of the morphogenetic programs of organs and maintains anatomical homeostasis. (Levin, 2021). Michael Levin suggested bioelectricity regulated by the transmembrane potentials can be viewed as the software to program the hardware of the cell to perform complicated transformations in morphogenesis. The transmembrane potentials of eukaryotic cells are regulated by ion channels and ion pumps; they both play important roles in embryo development and organ morphogenesis. Ion channels allow the ions to flow passively across the cell membrane down electrical and concentration gradients, whereas ion pumps transfer ions in reverse to the concentration gradients and are usually coupled with ATPase activities (Gadsby, 2009). Studies of the model organisms which included planarian, *drosophila*, zebrafish, *xenopus*, mouse, and human patients with specific genetic mutations of ion channels and ion pumps, suggested alterations of transmembrane potentials can cause dramatic morphological changes in animal development (Adams et al., 2016; Belus et al., 2018; Pai et al., 2018; George et al., 2019; Lanni et al., 2019; Levin, 2021; Pezzulo et al., 2021).

Ion pumps, including the plasma membrane variant of the V-ATPase proton pump, sodium/potassium ATPase, and calcium ATPase, are integral membrane proteins, and their activities are regulated by cytoskeleton proteins and microtubules through direct physical interactions (Devarajan et al., 1994; Arce et al., 2008; Ma et al., 2012). It is to be noted that a large proportion of the cellular ATP is consumed by the ATPases of ion pumps (Howarth et al., 2012). It is reasonable to assume the ATPases of ion pumps, which intrinsically associate with the cytoskeletons, serve as important energy sources for the dielectric oscillations of the cytoskeleton. Thus, the spatial and temporal alterations of transmembrane potentials of cells in an embryo are closely correlated with changes in dielectric oscillation frequencies of cells during embryo development. A recent study showed intermediate-frequency (100 kHz–300 kHz) electric fields altered the resting membrane potential of Hela cells as predicted by a Schwan-based mathematical model (Li et al., 2021). Consistently, Chang et al. have reported that intermediate-frequency electric fields (200 kHz) increased the membrane permeability of cancer cells (Chang et al., 2018). In the study, they observed an augmented number and sizes of holes on the cell membrane in glioblastoma cells when exposed to the EMF with scanning electron microscopy. Notably, exogenously forced alterations of membrane polarizations suppressed the malignant phenotypes of cancer cells (Brook Chernet, 2014). Thus, alterations of cellular transmembrane potentials may have direct impacts on the specific frequencies of cellular dielectric oscillations which further regulate gene transcriptions. The varied physical activities of ion pump ATPases and ion channels under different transmembrane potentials are likely to be involved in the relay of a chain of reactions.

Cellular electromagnetic fields are important in maintaining tissue homeostasis (Levin, 2021). The number of specific types of cells must be quantitatively regulated to embody a particular morphogenetic program or genetic traits. The electromagnetic field of the cells in a particular organ will instruct the stem cells and the progenitor cells when to differentiate or self-renew by interfering with the chromatin organization of these cells (Ross et al., 2015; Maziarz et al., 2016; Suryani et al., 2021). The subtle preferences over a particular cellular electromagnetic frequency to differentiate or remain quiescent for the stem cells would determine the size and the shape of a particular tissue. The regulatory loops involved in epigenetic changes of key transcription factors are triggered by the alterations of the electromagnetic oscillation frequencies of the surrounding chromatin regions of the transcription factors enriched with noncoding RNAs.

About 99% of the genetic information of the chromatin fibers is not transcribed. Studies indicate that these noncoding DNA sequences play important roles in regulating a broad spectrum of cellular functions (Statello et al., 2021). From the quantum biology point of view, these noncoding DNA sequences function as an antenna in the chromatin fibers to sense the variation of cellular electromagnetic fields (Zhao and Zhan, 2012b). Alterations of cellular electromagnetic fields change the oscillatory mode of these noncoding DNA sequences by directly interfering with the electromagnetic field of chromatin oscillatory subunits. Different electromagnetic oscillation frequencies and chromatin subunits would cause changes in chromatin organization accompanied by changes in epigenetic modifications and protein binding partners as described in the pulse couple oscillation clustering mode (Zhao and Zhan, 2012b; Manser et al., 2017).

Interestingly, the organs of metazoans are usually composed of different cell types, such as the liver is composed of hepatocytes, stellate fat-storing cells, Kupffer cells, and endothelial cells. Different cells are mixed together in close proximity in an organ, and differentiated cells are not likely to transdifferentiate from other cell types by the cellular electromagnetic fields of their neighbor cells. One possible explanation for this phenomenon is that the membrane potentials of cells from more differentiated states are usually hyperpolarized (Numaga-Tomita et al., 2019). Thus, they are much less sensitive to the environmental changes of EMF, and the intracellular EMF of differentiated cells is not likely to be interfered with by the alterations of EMF of other cells (Li et al., 2021). Alternatively, stem cells and progenitor cells usually possess depolarized membrane potential, which is more likely to transcriptionally respond to the changes of external EMF through the alteration of intracellular EMF (Levin, 2021; Li et al., 2021).

## Cellular electromagnetic fields in tissue damage repair and evolution

Metazoan species have to adapt to harsh environments that are constantly subjected to elements of destruction. The tissue-specific cellular electromagnetic field is an important factor in regulating the wound-healing system of a particular tissue (Saliev et al., 2014). Tissue injuries weaken the tissue-specific electromagnetic field of the damaged areas. Quiescent stem cells and progenitor cells residing in the vicinity of the injured area can directly sense the changes of tissue electromagnetic fields in their surroundings by their chromosomal oscillatory subunits. Thus, the external alterations of tissue electromagnetic fields can cause epigenetic changes in the progenitor cells surrounding the injured areas and further instruct the cells to proliferate and initiate the damage repair programs in the tissue (Ahmed et al., 2014).

Similarly, during evolution, environmental factors can constantly give feedback to a species on the size, along with other physical properties of a specific tissue, through damage-induced activations of the stem/progenitor cells. The activations of stem/progenitor cells alter the EMF and bioelectricity properties of specific parts of the animal body, which further leads to epigenetic changes in germ-line cells that are passed down to the offspring (Tseng and Levin, 2012; Durant et al., 2017). Thus, the alteration of the tissue-specific electromagnetic field is an important mediator within the chain of events leading to the adaptation and evolvement of specific morphogenetic features of a species (Tung and Levin, 2020).

## Cellular electromagnetic fields regulate cell migratory behavior

**FIGURE 4:**
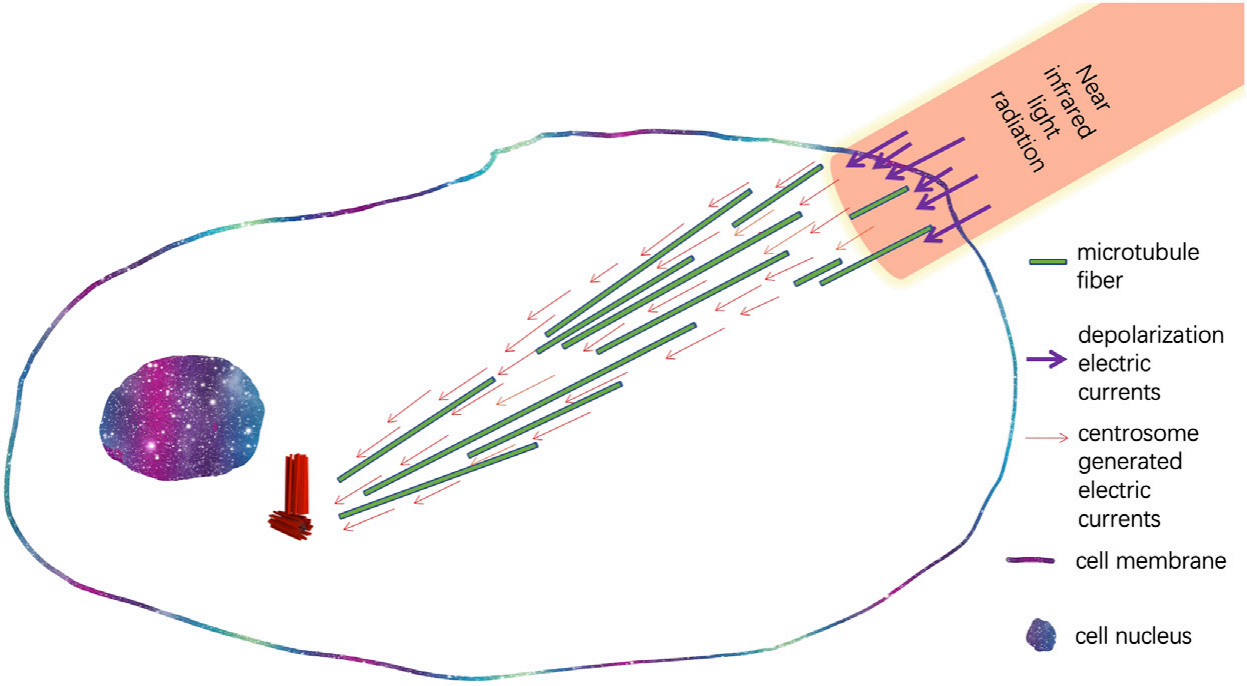
Illustration of synchronization of cytoskeleton electric currents originated from the centrosome with depolarized electric currents triggered by flashing infrared light.

In addition to transcriptional regulation, cellular electromagnetic fields also regulate the migratory behaviors of cells. Centrosomes can sense pulsating near-infrared light signals and can promote the projection of pseudopodia toward the light source, leading to the migration of cells toward the light source (Albrecht-Buehler, 1991; Albrecht-Buehler, 1994). Later, studies showed infrared light radiation is absorbed by the water molecules in the cytoplasm and increases the temperature of the local cytoplasm, which lead to the increase of electrical capacitance of the cell membrane and further induces depolarized electrical current near the radiated region of the cell membrane (Shapiro et al., 2012). Thus, an electrical circuit is formed between centrosomes and the infrared light-radiated region of the cell membrane, in which the depolarized electrical current connects with the electrical current generated by centrosomes through the cytoskeleton and cytoplasm. As nanoscale electrical pulses can trigger the assembly of microtubules, the electric currents lead to the directional growth of the microtubule network of cytoskeletons (Figure 4). Interestingly, in Dr. Albrecht-Buehler’s cell phototaxis experiments, the migration of cells is triggered by flashing light sources (Albrecht-Buehler, 1994). Flashing infrared light allowed periodical polarization and depolarization of cellular membrane potential, which periodically sustained the local depolarized electrical currents near the infrared regions of the cell.

It is noteworthy that heat-generating organs and the circulating blood of warm-blooded animals are able to emit infrared light signals (Kelly et al., 1954). The phototaxis migratory behavior of cells is triggered by flashing light sources with flashing periods of around one flash per second, which mimicked the rhythmic pressure changes of the cardiovascular system. The phototaxis behaviors of cells may help the cells to migrate toward the blood vessels and the heat source of organs, which would help the cells to obtain nutritional resources. Additionally, the thermogenic metabolism of cells can also generate near-infrared irradiation, leading to intercellular photonic communications between individual cells resulting in the aggregatory behavior of cells (Albrecht-Buehler, 1997; Albrecht-Buehler, 2005; Rahnama et al., 2011). Such a mechanism may help cells to maintain robustness in tissue regeneration and wound healing and also play an important role in organ morphogenesis.

## Cellular electromagnetic fields regulate neuron development

**FIGURE 5:**
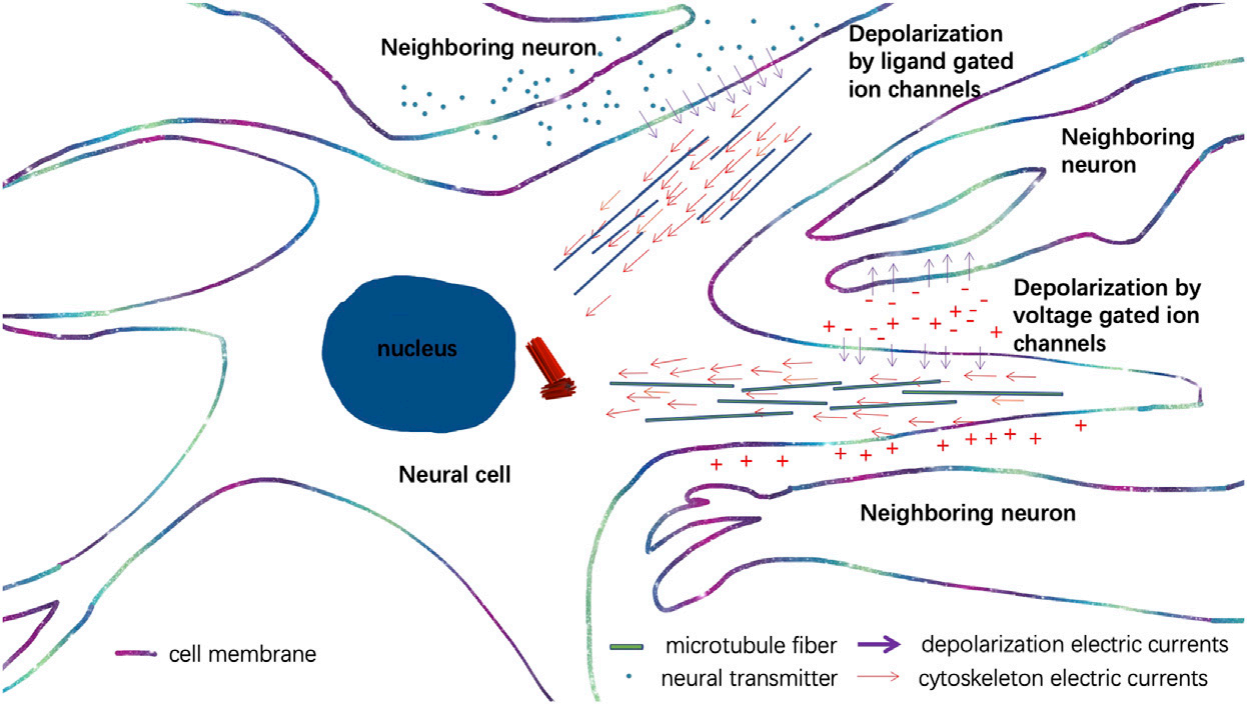
Illustration of the synchronizations of the centrosome and cytoskeleton electrical currents and depolarized electrical currents of neurons to direct the growth of neural tubes in a neural circuit.

Neural development of the brain is among the most challenging questions yet to be addressed in modern-day biology. Cellular electromagnetic interactions between different neural cells in a neural circuit play an important role in the morphogenetic program of forming and strengthening neural circuits (Cantero and Cantiello, 2020). A recent study suggested that neural electrical signals generated by the depolarization of the membrane potential can pass through the microtubule filaments of neural axons to provide another means of signal transduction which controls the exact timing for neuronal spikes (Singh et al., 2021). Thus, the electrical signals of nearby neurons and neural spikes mediate voltage changes in the proximity of the neural plasma membrane and can also trigger the directional projections of microtubule filaments in the neuronal axons orchestrated by centrosomes as discovered in Albrecht-Buehler’s experiment (Albrecht-Buehler, 1991) (Figure 5). Additionally, neurotransmitters also cause directional depolarization of neural plasma membrane triggered by ligand-gated ion channels of neurons, and the depolarized electrical currents can also synchronize with the cytoskeleton’s electrical currents generated by the centrosome, which further leads to directional growth of neural tubes and migration of neural cells (Figure 5). Such a mechanism orchestrates the self-organized autonomous morphogenesis programs of neural circuits.

## Conclusive remarks

Emerging evidence suggests the dielectric property of microtubules is essential for their functionalities and the nano-sized electromagnetic interactions are fundamental to the dynamic regulations of cell division and architecture of the microtubule-based cytoskeleton networks. Although the electrical generator model of centrosomes is supported by multiple lines of evidence, direct proofs for the model are still pending due to certain technical limitations. The microscopic understanding of the electromagnetic interactions within and between cells will provide us with a deepened knowledge of the self-organized mechanisms for organ development and morphogenesis from the embryo to neural biology. More detailed electromagnetic oscillatory models should be built for the already well-established electromagnetic oscillators, such as the chromosome and the microtubule. Novel electromagnetic oscillatory models should be conjured for various RNA and protein molecules. Electromagnetic oscillation as a ubiquitous physical phenomenon arches over every aspect of cell biology and is a phantom hand of art for classical molecular and cellular biology. From morphogenesis to functionalities of metazoan animals, the twilight of quantum biology may hold the key to addressing many challenging questions in biology and medicine.
